# Computed tomography/magnetic resonance imaging for mandibular boundary invasion of oral squamous cell carcinoma assessment

**DOI:** 10.1186/s12903-024-03920-8

**Published:** 2024-02-02

**Authors:** Yingding Ye, Xianglong Zheng, Tanhui Chen, Ke Zheng, Jie Pan, Lisong Lin

**Affiliations:** 1 Department of Oral and Maxillofacial Surgery, The First Affiliated Hospital, Fujian Medical University, Fuzhou, 350005 China; 2 Department of Radiology, The First Affiliated Hospital, Fujian Medical University, Fuzhou, 350005 China; 3 Department of Pathology, The First Affiliated Hospital, Fujian Medical University, Fuzhou, 350005 China; 4 Fujian Medical University, Fuzhou, China; 5 National Regional Medical Center, Binhai Campus of the First Affiliated Hospital, Fujian Medical University, Fuzhou, 350212 China

**Keywords:** Diagnostic imaging, Epithelial neoplasm, Fréchet distance, Mandible, Mouth cancer, Tumour boundary

## Abstract

**Background:**

The range of mandibular invasion by a tumour needs to be determined accurately to minimize unnecessary damage to the mandible. This study aimed to compare tumour boundary lines on computed tomography/magnetic resonance (CT/MR) images with those from pathological findings during the preoperative assessment of mandibular invasion by oral squamous cell carcinoma (OSCC). By comparing the methods, the potential of CT/MR for this application could be further elucidated.

**Methods:**

Eight patients with OSCC were imaged with CT/MR, mandibular specimens were collected, and the material site was measured. Haematoxylin–eosin staining was used for histopathological assessment. The presence and boundaries of bone invasion were evaluated. The CT/MR and histopathological boundaries of bone invasion were delineated and merged to compare and calculate the deviation of CT/MR and histopathological boundaries using the Fréchet distance.

**Results:**

The mean Fréchet distance between the CT and pathological tumour boundaries was 2.69 mm (standard error 0.46 mm), with a minimum of 1.18 mm, maximum of 3.64 mm, median of 3.10 mm, and 95% confidence interval of 1.40–3.97 mm. The mean Fréchet distance between the tumour boundaries on the MR and pathological images was 3.07 mm (standard error 0.56 mm), with a minimum of 1.53 mm, maximum of 4.74 mm, median of 2.90 mm, and 95% confidence interval of 1.53–4.61 mm.

**Conclusions:**

CT/MR imaging can provide an effective preoperative assessment of mandibular invasion of OSCC. Pathology images can be positioned on CT/MR scans with the help of computer software to improve the accuracy of the findings. The introduction of the Fréchet distance to compare tumour boundary lines is conducive to computer image diagnosis of tumour invasion of jaw boundaries.

## Background

Oral squamous cell carcinoma (OSCC) is one of the most common head and neck cancers and originates from oral mucosal epithelial cells with squamous differentiation. The tumour origin includes the buccal mucosa, gingival mucosa, posterior molar triangle mucosa, lingual body (anterior two-thirds of the boundary sulcus) mucosa, mouth floor mucosa, hard palate mucosa, and lip mucosa. Oral cancer markedly invades the surrounding tissues, and the range of invasion is closely related to cancer staging and prognosis. According to the American Joint Committee on Cancer staging system [1], when the oral tumour invades adjacent structures only (e.g., through cortical bone of the mandible or maxilla or through the maxillary sinus or skin of the face), it can be clinically diagnosed as a moderately advanced tumour (T4a). Moreover, superficial erosion of bone/tooth socket (alone) by a gingival primary is not sufficient to classify a tumour as T4.

Mandibular invasion is a common occurrence and influences patients’ prognosis [2, 3]. Currently, the molecular mechanism by which OSCC invades the mandible is unclear. OSCC may interact with cells such as osteoblasts and osteoclasts at the anterior of the tumour (tumour–bone interface), providing a microenvironment conducive to osteolysis and causing damage to the jaw. The growth factors within the jaw can also promote tumour cell growth, further increasing the tumour’s invasive ability [4]. Therefore, if mandibular invasion by oral cancer is clinically diagnosed, the involved region of the mandible needs to be resected along with the tumour [5]. The method used for surgical resection depends on the range of mandibular invasion by the tumour and may include segmental osteotomy or marginal osteotomy of the mandible [6, 7]. After a large amount of tissue has been removed, reconstructing the facial tissue is often required. Reconstruction can be performed using flaps which match the skin aesthetic, such as the deltopectoral flap [8].

Computed tomography (CT), magnetic resonance imaging (MRI), and ultrasound are commonly used imaging methods [9, 10]. With advances in CT and MRI resolution, digital preoperative planning has also become routine. Intraoperative guides made according to the pre-determined extent of surgical excision and the size of the tissue required for reconstruction help reduce the difficulty and the time required for the procedure. Reduced surgical duration is beneficial for the range of surgical indications and the tolerability of surgery for the patients. Therefore, the range of mandibular invasion by the tumour needs to be determined accurately to minimize unnecessary damage to the mandible. Advancements in ultrasound technology have allowed for direct tumour assessment, and various studies have explored ultrasound’s applicability for evaluating OSCC [11]. However, accurate measurement of the tumour boundary on CT/MR images is clinically required [12]. Although numerous studies have assessed whether the tumour invades the mandible, retrospective studies cannot compare the imaging and tissue pathological results [13]. Therefore, this study aimed to compare tumour boundary lines on CT/MR images with those from pathological findings during the preoperative assessment of mandibular invasion by OSCC. We conducted an isoplanar fitting of pathological, CT, and MR images and then measured the Fréchet distance, a measure of the Euclidean distance between two irregular, unequal curves, of the boundary of mandibular invasion by OSCC.

## Methods

This study observed 33 patients with OSCC from The First Affiliated Hospital of Fujian Medical University from March 2021 to December 2021. This study was approved by Branch for Medical Research and Clinical Technology Application, Ethics Committee of First Affiliated Hospital of Fujian Medical University, China. The study was conducted in accordance with the ethical standards of the Declaration of Helsinki. All participants signed an informed consent form after receiving a detailed explanation of the study.

### Inclusion criteria

Patients were included if they had OSCC involving the mandible, as evidenced on CT/MR; if they were undergoing the first surgery for OSCC without preoperative radiotherapy or chemotherapy; if the surgery included complete excision of the primary site, partial mandibular excision, and cervical lymph node dissection (with or without vascularized free flap graft repair); and if they had complete clinical history, pathological data, and imaging data available.

### Exclusion criteria

Patients were excluded if their cancer did not involve the mandible; they had recurrent tumours; they had undergone radiotherapy; they had metastatic cancer; or their pathological images and imaging data were incomplete or unavailable.

### Image acquisition parameters

Every patients underwent both CT and MR. MR images were obtained from all patients with a Verio 3.0-T scanner (Siemens Healthineers, Erlangen, Germany), and head and neck coils were attached. Scan parameters were as follows: spin echo T1-weighted imaging; turbo spin echo T2-weighted imaging; and 5-mm thickness, field-of-view 22 cm × 22 cm. The contrast agent for the enhanced scan was a gadopentetate glucosamine dose of 0.1 mmol/kg.

CT images were obtained using the Aquilion 64-row 128-slice helical CT system (Canon Medical, Otawara, Japan), voltage 120 kV, current using intelligent tube current regulation technology, 0.5-seconds rotation speed, 1-mm slice thickness, 0.8-mm slice spacing, 512 × 512, isotropic, and evaluation using a bone window.

### Biopsy localisation

**Fig. 1:**
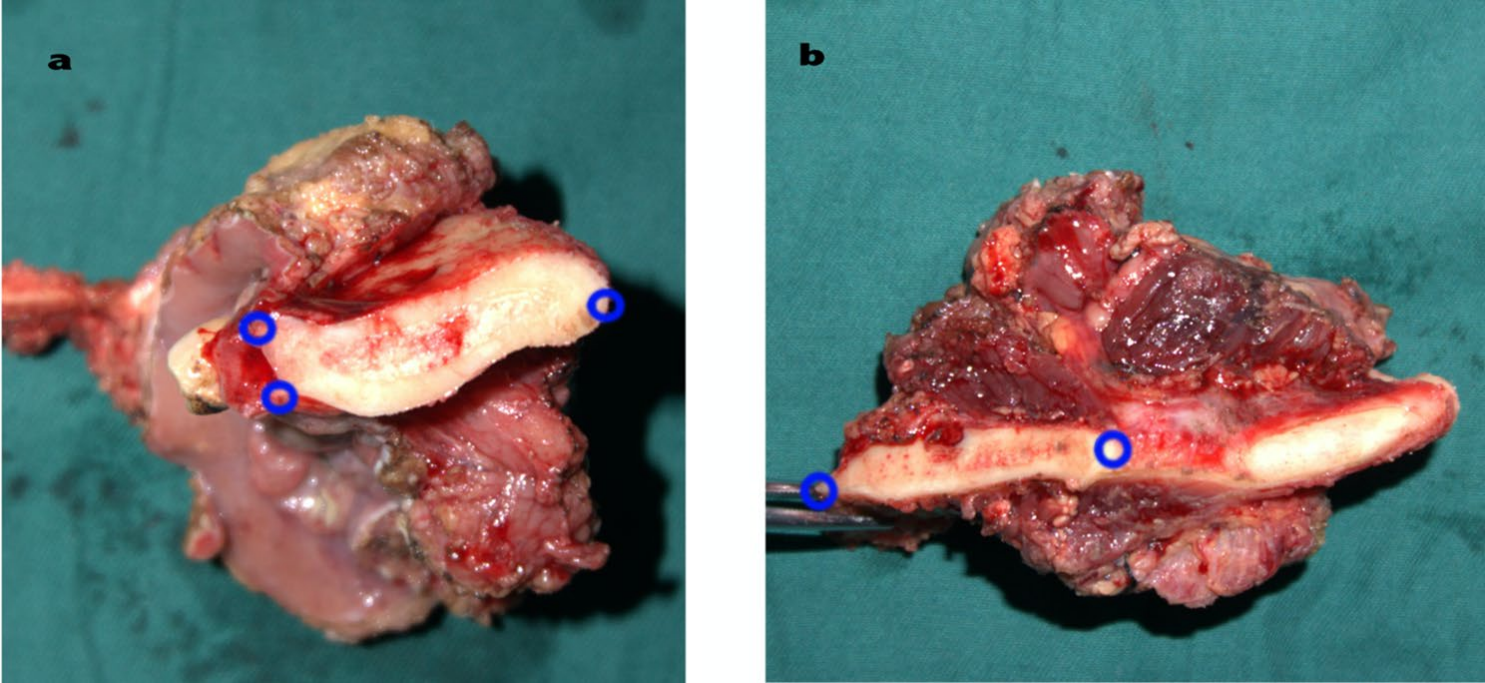
The ex vivo bone specimen. **a** Near the middle of the ex vivo bone specimen, the blue circle is the selected measurement position. **b** The distal mid-end of the ex vivo bone specimen is marked by the chosen measurement location

The resected mandibular specimen was dissected into two parts using a chainsaw/surgical power system at the deepest point of suspected tumour invasion, along the centre of the tumour. After ensuring hard tissue biopsy sectioning, the length of the mandibular biopsy section from the lower edge of the extraction section, superior margin buccal, and lingual sides were measured using a dividing gauge and a straight-edge. The corner formed by the section and the bone surface was used as a marker (Fig. 1), and anatomical markers could also be used for marking. The mean of two measurements was used, and then sectional bone specimens were divided again at approximately 3–5 mm.

In the process of biopsy measurements, this study used a parting gauge and a straight edge to measure the markers using the corner formed between the section and the bone surface after osteotomy. In the actual measurement process, the points selected were recorded, and the outermost corner point of the buccal side, the outermost corner point of the lingual side, and the point at the lowermost edge of the mandible were used to determine the biopsy plane. Before measuring the section, the mandibular specimen was first isolated and then positioned, after which the biopsy section was removed. This was done to avoid inaccurate positioning due to the loss of the mandibular specimen caused by the destruction of the saw blade when the mandibular biopsy is resected, which usually results in a loss of 1–2 mm of thickness in the mandible.

During the process of making pathological sections, the orientation of the specimen on the original jawbone was reaffirmed before specimen embedding. The location of the starting section was determined, and the location of the specimen used was recorded during sectioning. With imaging accuracy of 1-mm CT slice thickness and 0.8-mm slice spacing, the bone specimen thickness was 3–5 mm. After localising the biopsy plane on the reconstructed image, approximately 2–3 levels were involved on CT, and 1–2 levels were involved on MRI; moreover, there was no need to switch the imaging level when the tissue loss was within 2 mm.

### Processing of medical images

**Fig. 2:**
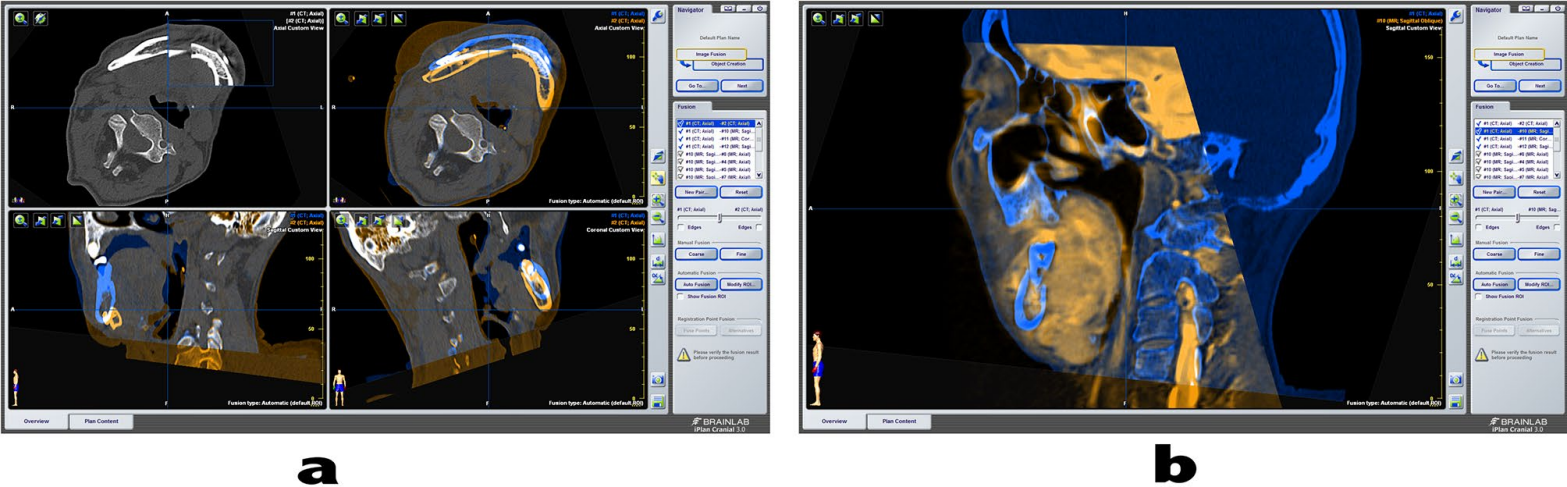
Image fusion is performed by computational software. **a** The preoperative and postoperative CT images were fused to show that the position of the mandible was different. **b** CT and MR image matching fusion

**Fig. 3:**
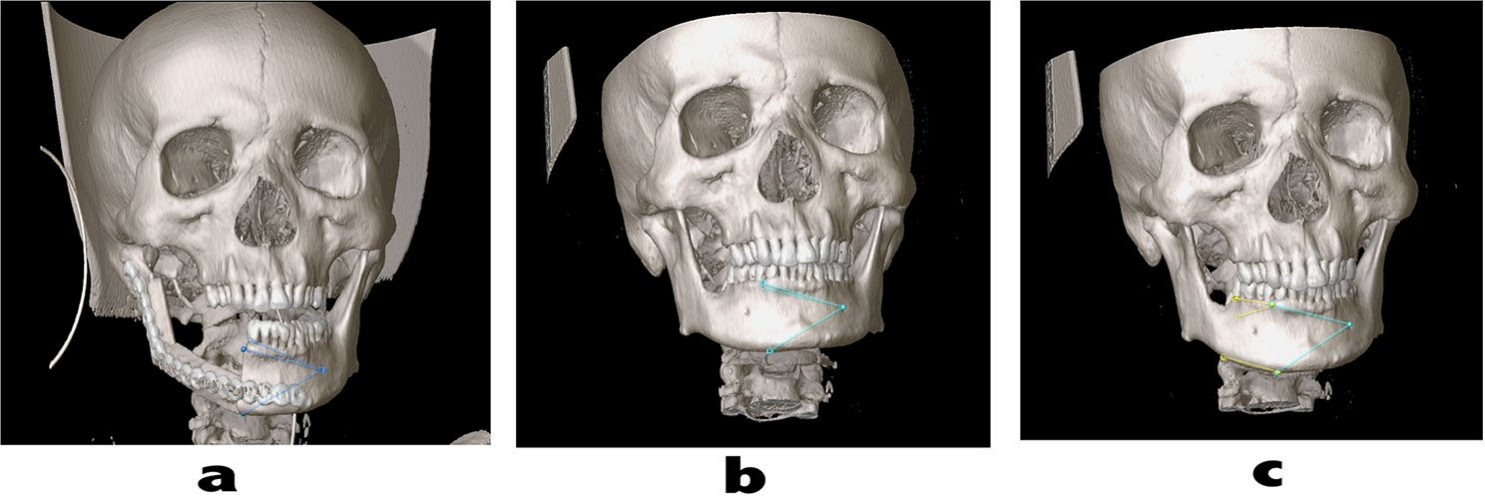
The pathological sample was located on the computerized reconstruction image. **a** The osteotomy location is marked on the postoperative CT image, and the position relationship between the points and the chin foramen is recorded. **b** The position relationship between each point and the chin foramen was used to correct the position deviation of the mandible, and the osteotomy position was located on preoperative CT. **c** The yellow path points to the material location

Patient’s preoperative and postoperative imaging data (CT/MR) were retrieved from the picture archiving and communication system. On the Image Fusion interface of the BrainLabiPlansoftware (https://www.brainlab.com/radiosurgery-products/iplan-rt-treatment-planning-software/download-iplan-rt-4-5-7-viewer/), preoperative and postoperative CT images were merged to match the images of the patient’s skull base and maxillary structures. The preoperative MR and CT images were fused using the same method (Fig. 2). By positioning the measured value at the time of material acquisition to the material site on the CT/MR image (Fig. 3), we could obtain a CT/MR image in the plane of the material. According to the scale of CT/MR images, the pathological images were appropriately reduced in size and matched with the reference to locate the centre point and shape.

### Criteria used for outlining the tumour border

Then, two oral and maxillofacial surgeons outlined the tumour border, which was reviewed by one senior imaging specialist and two pathologists. Criteria for creating the border outline were as follows [14]. For CT, the criteria were discontinuous bone cortical defects due to adjacent tumours; destruction of the bony trabeculae and invasion of the cortical bone; and morphological disruption of the mandibular nerve canal. For MRI, the criteria were the replacement of the T1/T2 low signal intensity of the bone cortex by the high signal intensity of the tumour; low T1 signals and high T2 signals of bony trabeculae in enhanced images; and high T1-weighted signal under the enhancement of the mandibular nerve canal adjacent to the tumour.

Tissues without tumour cells in the tissue structure at the anterior of tumour infiltration were used to outline the border of the tumour on pathological images.

### Mapping of tumour outlines

This study used Python to write a function to read curve coordinate sets inversely from tumour border outlines drawn by the mapping software. Because this study involved multiple curves fitted to multiple images, the pathological boundary was labelled green, the MRI tumour boundary was shown in red, and the CT tumour boundary was shown in blue to distinguish them. Using the tumour boundary outline image after fitting, the pre-set Python programming code was retrieved, and curve coordinate sets were read using different colours.

Based on the curve coordinate sets read, a recursive method was used to calculate the Fréchet distance between every two curves.

### Statistical methods

General count data were described statistically. Descriptive statistics of measurement data were obtained for the Fréchet distance between the tumour boundaries on CT and pathological images, as well as for the tumour boundaries on MR and pathological images, including the mean, minimum, maximum, median, and 95% confidence interval. Statistical analysis was conducted with SPSS Statistics v25 software (IBM SPSS, Inc., Armonk, NY, USA).

## Results

### General data

We observed 33 cases of partial mandibular resection for oral cancer surgical treatment, of whom 13 cases were clearly pathologically diagnosed with mandibular invasion of oral cancer. Of these, four cases lacked complete biopsy acquisition data and pathological images of mandibular invasion by oral cancer, one case was a recurrent tumour, and one case could not be fitted to the pathological images. Thus, these individuals were excluded. The remaining eight cases were collected as case studies, among which three cases only had CT image studies because of preoperative intraoral metallic dentures affecting MR images. The eight individuals comprised seven cases of gingival carcinoma and one case of mouth floor carcinoma. Patients’ general characteristics are shown in Table 1.

**Table 1:** General characteristics of the cases

Data Classification	Classification Type	Count Results
Sex	Male Female	5 (62.5%) 3 (37.5%)
Age (years)	>= 60 < 60	5 (62.5%) 3 (37.5%)
Primary Site	Floor of the Mouth Gingival	1 (12.5%) 7 (87.5%)
Tumour stage	T3 T4	1 (12.5%) 7 (87.5%)
Lymph node metastasis	Yes No	5 (62.5%) 3 (37.5%)
Maxillectomy method	Segmental resection Marginal resection	6 (75.0%) 2 (25.0%)
Type of Mandibular invasion	erosive infiltrative	4 (50.0%) 4 (50.0%)

### Assessment of tumour boundaries

**Fig. 4:**
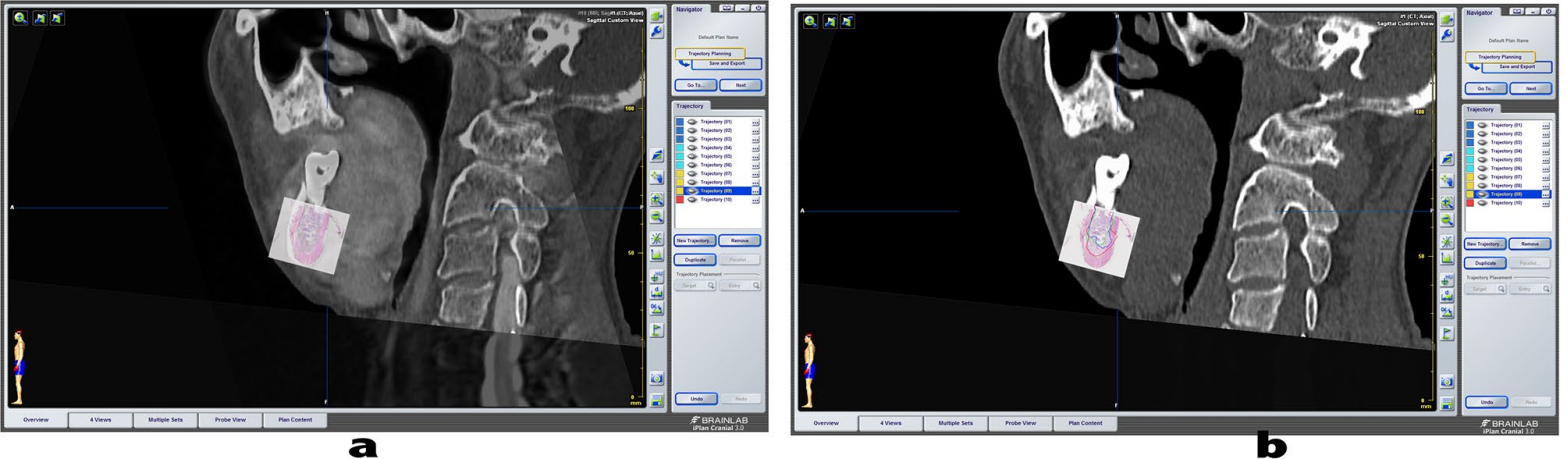
Locating pathological images in CT/MR images and performing image fusion. **a** The pathological images were scaled down and fused with CT/MR images. **b** Mark of the respective tumour boundaries on the fused images

The mean Fréchet distance between tumour borderlines on CT and pathological images and the mean Fréchet distance between the MRI and the pathological tumour borderlines are shown in Table 2. We used the Frėchet distance tool on the computer software to measure the distance on the curve on the images, and the resultant Frėchet distance was calculated in pixels, and after the unit conversion, the accuracy of the Frėchet distance measurement was 0.01 mm. Figure 4 shows the tumour boundary after CT/MR and pathological image matching.

**Table 2:** Assessment of tumour boundaries

Data Classification	Fréchet distance (mm)	standard error (mm)	Minimum (mm)	Maximum (mm)	Median (mm)	95% confidence interval (mm)
CT/pathological	2.69	0.46	1.18	3.64	3.10	1.40–3.97
MR/pathological	3.07	0.56	1.53	4.74	2.90	1.53–4.61

## Discussion

In this study, we used postoperative CT and MR images of the jaw bone defect area of patients with OSCC to define the landmarks of the mandible, and then moved markers to the preoperative images. After continuously comparing numerous layers of the reconstructed CT sections, the imaging location found could be considered similar to those provided in the pathological images, and positionally reconstructed image sections had high similarities. Comparatively, the MR images consistently overestimated the boundaries of the pathological image features. These findings suggest that CT images may be used for preoperative assessment of mandibular invasion of OSCC. Our ultimate goal was to provide findings which could enable preoperative diagnosis of CT/MR tumour boundaries via computer software and to use the Fréchet distance to correct diagnostic criteria. By using computer software and the defined diagnostic criteria, the process could readily facilitate machine learning, potentially providing rapid and accurate diagnosis for future patients.

The Fréchet distance is a description of the path and spatial similarity developed by French mathematician Maurice René Fréchet in 1906; the definition of Fréchet distance is as follows: the shortest maximum distance between two curves with directions that cannot be traced. His description also considers factors of distance between path and space [15]. We believe that the direct calculation of the distance between the two tumour boundary curves is beneficial for artificial intelligence to identify and diagnose them [16].

**Fig. 5:**
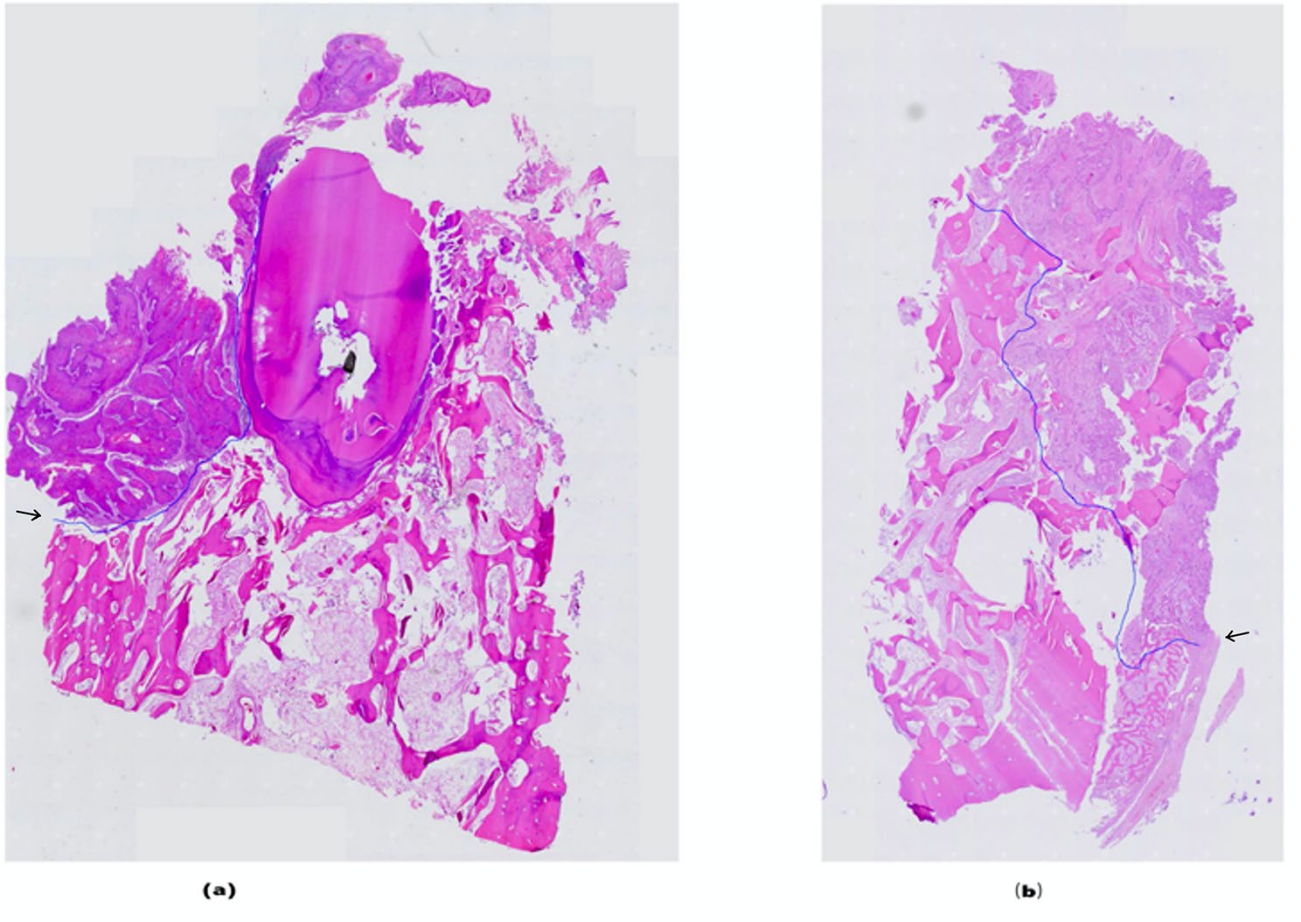
Two different types of images of jaw pathology invaded by tumours. Black arrows indicate two curves respectively. **a** Approximate U-shaped mandibular invasion by oral squamous cell carcinoma at 20× magnification; the blue line suggests the extent of the tumour. **b** Irregular mandibular invasion at 20× magnification; the blue line suggests the extent of the tumour

Traditional evaluation of the depth of tumour infiltration involves connecting the lines between the tumour and nearby normal tissue boundaries and measuring the perpendicular distance between the connected line and the deepest tumour infiltration [17]. This measurement method is particularly applicable to U-shaped tumour infiltration types (Fig. 5a). If the tumour has an irregular, multidirectional infiltration (Fig. 5b), the normal tissue boundaries on the two sides are difficult to determine. Clearly, the traditional method cannot accurately evaluate the depth of infiltration. Due to the different image production principles of CT and MRI, images at the boundary of the tumour-infiltrated mandible will vary markedly. For tumour boundaries that form an irregular curve, the length, range, and shape may differ markedly across images. It is difficult to make appropriate comparisons between image boundaries using the traditional evaluations of the depth of tumour infiltration.

On the pathological image, we observed that a small part of the bone invaded by the tumour retained high mineral density; however, the density did not appear to be retained in the image provided by CT. Conversely, the part of the bone that was not invaded by the tumour exhibited a decrease in mineral density on the pathological image and a lower mineral density was also visible on the associated CT image. To explain this discrepancy, we hypothesize that some intermediate mediators may facilitate the destruction of the jaw by a tumour and that osteogenesis and osteoclast are present at the same time. Further experiments with larger sample sizes are needed to validate these hypotheses.

The tumour boundaries were consistently overestimated in MR images. Despite MRI tumour boundaries being less comparable to the pathological results than that of the CT images, it effectively portrayed the lesion range of the tumour and the tumour front, which led to some overestimation of the boundary evaluation. This finding aligns with the traditional understanding of MRI and should be taken into consideration in future studies.

The tumour boundary line outlined on the CT image actually indicated the border of mandible resorption caused by tumour invasion. We speculated that the reasons for CT overestimation and underestimation of mandibular invasion by the tumour were as follows. First, in the case of CT boundary underestimation, the edge of the mandibular tissue has been invaded by tumour tissue, but the degree of bone breakage and decalcification in this area has not reached a level that can be recognized by human eyes on the CT image. Alternatively, the osteogenic repair of jawbone tissue is faster, to the point that the mandibular density has not rapidly decreased. Second, in the case of CT boundary overestimation, the edge of mandibular tissue has been invaded by tumour tissue, which has caused a more intense inflammatory reaction, resulting in rapid destruction of mandibular tissue and a rapid decrease in mandibular density. Thus, the extent of mandibular resorption will be greater than the extent of tumour invasion.

Using the above analysis, the boundary lines of tumours on CT and MR images and pathological images were reassessed. In cases where the CT and pathological tumour boundaries were very similar, we observed that some CT boundaries were overestimated while others were underestimated. Thus, it could be determined that simple overestimation and underestimation using the full line segment could no longer satisfy the evaluation of tumour boundaries. For example, in the upper segment of the buccal tumour boundary line, there is a possibility of overestimating the CT tumour boundary line. In the buccal middle and lower segments, underestimation of the CT tumour boundary line could occur. In the lower bottom-end segment, again, there may be an overestimation of CT tumour boundary line. We speculate that, in the region of the jaws where the tumour invades the front, part of the immune response causes more severe destruction of the bone tissue, while another portion causes more rapid repair of the bone tissue, albeit with some tumour cells present within it. Such a dynamic understanding of the tumour borderline could better explain the appearance of both types of manifestations of erosion and infiltration on imaging.

This study presented a simple and effective method of biopsy localisation and met clinical specifications. The computer-reconstructed biopsy section and pathological images had similar localisation and a high matching score. This method can be used for any specimens with relatively little deformation of isolated tissues, such as typical bone tissues. Follow-up studies can use adjusted values based on CT/MRI imaging evaluation of tumour boundaries, find safe surgical margins at the tumour boundaries, and resect the mandible strictly following a digitalized surgical plan to have ideal results for precision surgery. These studies will mark regions of interest on preoperative images, conduct biopsies after performing localisation on the mandible specimens, and enable pathological diagnosis of precise areas.

This study developed a method for the evaluation of tumour boundary lines using the Fréchet distance. Based on its principles, curve evaluation could avoid the effect of curve shape on objective judgment and make use of computer programming logic. This approach could be used for AI diagnosis and can be extended from 2D images to 3D images for tumour boundary evaluation.

This study had some limitations. First, the sample size was very small. The limited sample size arose due to the exclusion criteria employed and the single centre approach. Future studies should ensure an appropriate sample size and multi-centre design are used to improve the generalisability and value of the findings. Second, boundary fitting using morphological similarities has a relatively high requirement to produce pathological specimens. In the production of pathological specimens, tissues are unavoidably lost and ripped, which reduces the accuracy of such fitting. In the processes of biopsy and production, the requirements for the integrity of the tissue morphology are higher.

## Conclusions

Through the positioning and measurement of pathological materials, the CT image of the postoperative mandible could predict the preoperative CT/MR image, whereas the tumour boundaries were consistently overestimated in preoperative MR images. Therefore, in the clinical setting, CT imaging could preoperatively verify the diagnosis and determine the scope of osteotomy for OSCC. Using the images, the preoperative surgical plan can be planned in detail, thus improving the potential surgical outcomes and quality of life of the patient. Specifically, the tumour can be completely removed using surgical guide and navigation equipment and the jaw tissue can be preserved to the greatest extent. The introduction of the Fréchet distance to compare tumour boundary lines is conducive to computer image diagnosis of tumour invasion of jaw boundaries. The developed approach could be used for AI diagnosis and can be extended from 2D images to 3D images for tumour boundary evaluation.

## Data Availability

The data that support the findings of this study are available from the corresponding author, but restrictions apply to the availability of these data. Data are, however, available from the authors upon reasonable request.

## Abbreviations

CT: Computed Tomography
MRI: Magnetic Resonance Imaging
OSCC: Oral Squamous Cell Carcinoma
